# Proposed Alterations at the Oxford Infirmary

**Published:** 1891-08-01

**Authors:** 


### PROPOSED ALTERATIONS AT THE OXFORD INFIRMARY.

### THE SHAKING OF THE DRY BONES.

OUR Special Commissioner has not borne the brunt of the storm in vain. At the quarterly meeting of the Oxford Infirmary Committee on Wednesday last, it was admitted that it had become impossible to ignore the just criticisms made upon the institution in the pages of THE HOSPITAL. After much discussion a resolution to this effect was put and carried: "That a new male ward be erected; that money be raised for this purpose; and that the old block should be rearranged for administrative purposes." It is proposed to devote the sum of £6,000 to the erection of the new building and £4,000 to the renovation and alteration of the old. Whilst we fully concur in the necessity of new wards, we think the retention of the present main building of doubtful advantage. Four thousand pounds would not go very far towards making such an ancient fabric substantial and complete. This antiquated building was condemned even in the last century on structural grounds. We trust that the present committee will prove themselves desirous of erasing the failures of the past, and that another seven or eight years will not elapse ere they are in possession of an institution worthy of themselves and Oxford. With Mr. Waddilove's munificent offer before them they cannot complain of want of encouragement from without. We should be more hopeful if the attendance of Governors displayed a little vitality. As it is the Governors of the Oxford Infirmary are conspicuous only by their absence from the quarterly meetings.

